# Supplementary material for: The causal relationship between the human gut microbiota and pyogenic arthritis: a Mendelian randomization study
Source: Front Cell Infect Microbiol. 2024 Nov 26;14:1452480. doi: 10.3389/fcimb.2024.1452480 (PMC11629706; doi:10.3389/fcimb.2024.1452480)
Supplement: Supplementary file 1 [file DataSheet1.zip › mendelian test/ebi-a-GCST90027768.csv_leave_one_out.pdf]

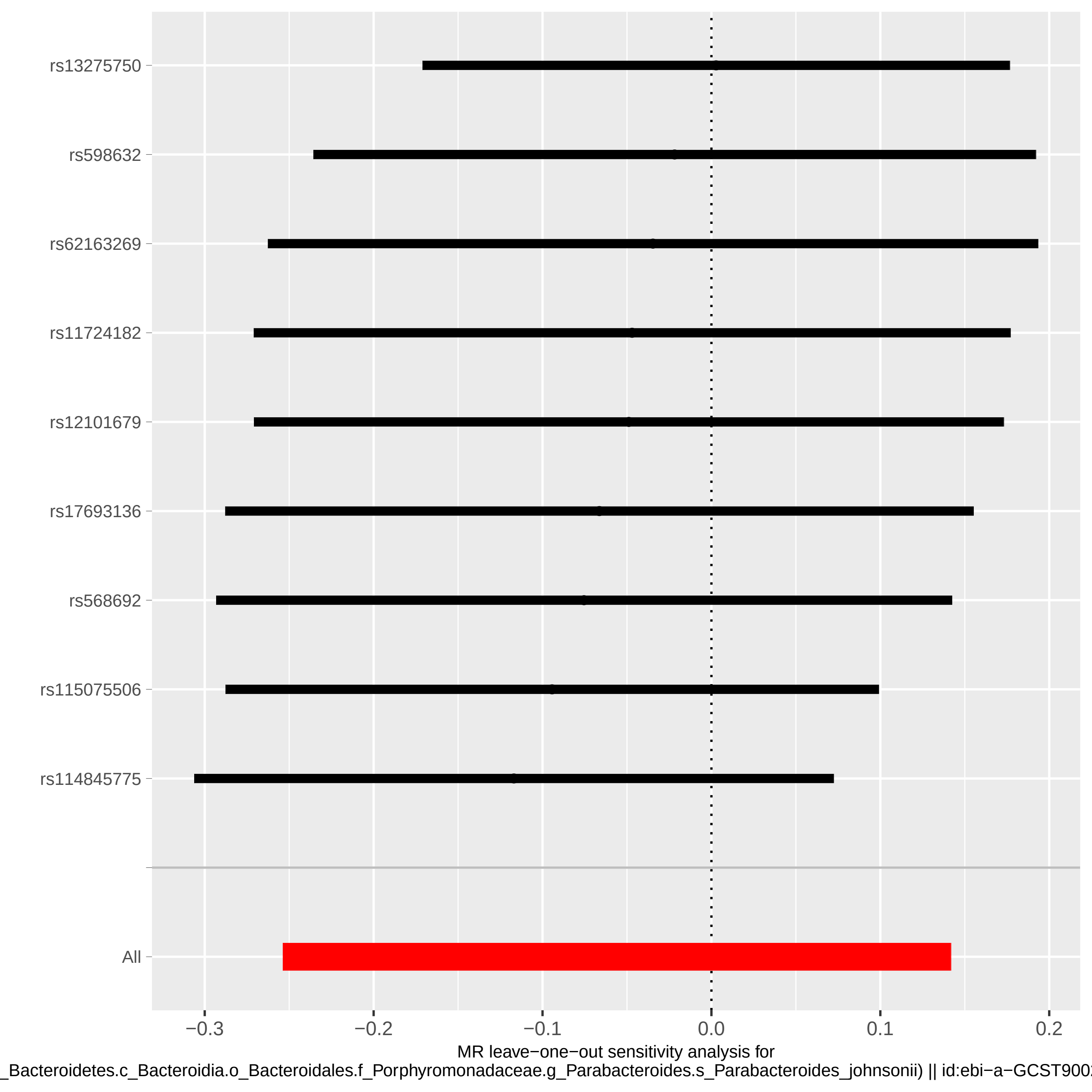
rs13275750
rs598632
rs62163269
rs11724182
rs12101679
rs17693136
rs568692
rs115075506
rs114845775
All
−0.3
−0.2
−0.1
0.0
0.1
0.2
MR leave−one−out sensitivity analysis for
_Bacteroidetes.c_Bacteroidia.o_Bacteroidales.f_Porphyromonadaceae.g_Parabacteroides.s_Parabacteroides_johnsonii) || id:ebi−a−GCST900
